# Foreign Correspondence

**Published:** 1873

**Authors:** 


### FOREIGN CORRESPONDENCE.

VIENNA, July 10, 1873.

*My dear Dr. Gaillard:*

In all my previous visits to the Old World, the interests of my beloved profession have always claimed my close attention. My first embraced a period of over two years, during which occurred the revolution of the three days of July, in Paris, in 1830, and in November of that year Poland attempted to throw off the oppressive yoke of Russia. As the gallant Pulaski fell at the siege of Savannah, in my own native State, during the Revolution of 1776, (and no man knoweth of his grave to this day,) I felt called upon to offer my feeble services in her behalf. Not knowing the language, I served as surgeon to a hospital in Warsaw, where, having obtained the Cross of honor, was promoted to Surgeon Major of the fifteenth regiment, and chief of the ambulances attached to General Turno's division. When Warsaw fell by assault, 7th of September, 1830, I was imprisoned by Prussia for thirty days, under the plea of cholera, and did not reach the United States until 1832. It was during this sojourn in Europe that I met Sir Astley Cooper, Mr. Abernethy, (afterward Sir William Lawrence,) and others of London ; Mr. Carmichael, Mr. McNamara, of Dublin; and Larrey, Dupuytren, Boyer, Richerand, Roux, Trousseau, Rostani, and at subsequent occasions, Nélaton, Velpeau, Ricord, Larrey, the son, etc., etc.

Last October, in rapidly descending a stairway, the heel of my boot caught on a step, and I was pitched forward and downward some eight or ten feet. The railing of the banister was grasped at, but not seized. I was momentarily conscious of what a terrible blow was to be received, threw up my hand to protect the face and head, and in an instant afterward, was insensible. The last recollection—and that not very distinct—was the sound made by striking the floor. Recovering in a few moments, and remembering the excellent advice of my friend, Professor Hodgen, of the St. Louis Medical College, in an article published on the treatment of recent injuries, not to raise or disturb a patient too soon when the brain was disordered, I plead with my family and friends not to lift me up, but simply to straighten out my limbs. The principal injury sustained was partial paralysis to the soft palate, due, as was supposed, to the fact that the resistance offered by the hard floor (not carpeted) passing through the uplifted hand, cranium, brain, and palatine bones was expended on the soft, floating palate. I could operate, did even perform lithotomy before the class, but could not lecture; and experienced difficulty in swallowing food not well insalivated or moistened. This, with some contusion on the wrist, and occasional pain behind the right ear and down the neck (the face being turned, at the fall, to the left side) with some slight disturbance of the brain, were all that I had hoped would remain to remind me of the dreadful blow. It is probably right to a proper understanding of the case—though I dislike to write so much about myself—to state that my weight is about two hundred pounds, am over six feet high, sixty-seven years old, born on a rice plantation, never used whisky or tobacco, and have scarcely ever been sick.

At the close of the last course of lectures, after a very hard day's labor and much mental excitement, while standing up giving directions to the son, a colored man, whose mother's breast had been removed before the class, I suddenly lost all consciousness and fell, in a fainting state, upon my face. It was this which induced me to seek change and relaxation by this seventh visit to Europe. So far, however, the care and anxiety of a large party leave but little time to devote to hospitals, visiting professional friends or collating medical facts. Indeed, this letter is one of apology for any expectations you may have had (the right it may be) created, by my name being published as a collaborator to your excellent journal; now acknowledged to be the best monthly in the profession. Traveling with eight ladies, and but one of them comprehending but a single foreign language, I am in the position to appreciate the humane decision of the Irish Judge, who, when the bigamist, after conviction, was proven to have a third wife, let him go free, proclaiming that that was too much for any mortal man.

In order to be certain that there should be no exposure to any one under my care taking cholera, I wrote, in advance, to Professor Theodore Bilroth, U. D., and recived a prompt answer while in Munich. In calling upon him, soon after our arrival, we found him hard at work by gaslight, though it was not yet quite dark.

Yesterday, at the Exposition, Dr. Taylor, the Orthopedist of New York City, (whom I met in Paris in 1867, where he obtained the prize for his method of treating spinal affections,) met the professors of surgery and others of this city. There, in a public hall, what he explained in English, Dr. Bilroth translated in German for his friends. I am pleased to state our countryman made a most favorable impression and secured the hearty commendation and thanks of all present. What struck them most was the facility with which, by a simple key, the same apparatus was made to fit almost any child. When some objected to the chinpiece, in diseases of the cervical vertebræ, preventing the patient taking food, and Dr. T. showed how the alligator opened his mouth, by elevating the superior maxillary, which Dr. B. translated crocodile, there was great satisfaction expressed, and quite a laugh at the expense of the objectors.

Dr. Bilroth is now forty-four years old, is a large, powerful, and very handsome man, in the prime of life; speaks English well, has a fine voice, easy flow of language, and is no doubt eloquent, on occasions. He came out of the late European war with more character than any European surgeon, and, in my humble judgment, is the *first surgeon now alive.*

Yours truly, Paul F. Eve, M.D.

---

Paris, Agust 7, 1873.

*My dear Friend, Dr. Gaillard:*

Just as I supposed when I wrote you from Vienna, and where we found the imposition about equal to the Exposition, grand as that may have been, such has been my constant occupation, that no medical notes could be written out. Should we have a good time at sea, on our voyage home, it is possible that something more will be attempted for the Journal. At present, it is proper only to give a brief notice of what has been seen to-day.

Called, by appointment, upon Dr. J. Péan, Chirurgeon des Hôpiteaux de Paris, with my son Duncan, student of medicine; we were taken by him, in his carriage, beyond the walls of Paris to his Maison de Santé, to witness, or assist, as the French say, in an operation for removal of the uterus. The patient was a large, fleshy woman, about fifty years old, who for years had laboured under a large abdominal tumor. It presented nearly the size at the full term of utero-gestation, projected more to the right than the left side, and reached above the umbilicus several inches. It was diagnosed to be of the fibro-cystic class, such as contained thick, viscid fluid.

The patient was placed on a narrow table of the Doctor's own construction, the abdomen exposed, and urine drawn off. The operation was seated between her thighs, with half a dozen assistants, besides the one who administered chloroform, the anæsthetic exclusively used in this hospital. This was sprinkled from time to time on lint covered on one side by oil-silk. The pulse was not felt once during the two hours the operation lasted, but the respiration was attentively watched. This was well managed. Although the day was not cold, the sash was kept down, a door alone left open, and yet the room was small and occupied by some twenty persons. I never saw a greater number of instruments used in any operation, or the general preparation for one more complete. The *chirurgicum armentum* was immense.

The operation was well done, notwithstanding its duration. The line of incision was traced by ink, and extended from two inches above the umbilicus to the pubis. The dissection down to the peritoneum and exposure of the tumor was made by a master hand in surgery. The greatest care was exercised in preventing blood getting into the abdominal cavity, or the loss, unnecessarily, of a single drop of it. These struck me as the two great points in the operation—namely, to check hemorrhage and avoid all sources of peritonitis. In delivering the tumor, the opening made had to be enlarged by scissors, while the mass was transfixed by very long needles, through the eyes of which strong wires were deposited in different directions, and these drawn upon pretty forcibly by assistants. It required considerable force to be extracted, and then laid upon the now flaccid walls of the abdomen. The cysts contained chiefly gelatinous matter, not fluid enough in some of them to readily escape; so that punctures would not have much diminished the size of the growth. It proved to be a fibrous degeneration of the womb itself, involving the whole of the body of this organ, leaving only the os and neck unaffected. The ovaries with the fallopian tubes were also removed.

Another peculiarity of Dr. Péan's method is, that he does not excise the diseased structures at once, nor divide the parts by an écraseur, but cuts its way piece by piece, like the cutting off of beef-steaks; previously, however, preventing bleeding by the stout steel wires, already alluded to, placed under the incisions and drawn very tightly by strong serre-nouds, knot-tighteners. In this way the pedicle of the tumor was diminished to about an inch, over which the sheet-lead was placed, having an opening in its centre, and the whole secured by a bandage. The wound in the abdominal wall was closed by alternately applying the interrupted and twisted sutures.

By a number of broad-bladed forceps, as soon as the peritoneum was laid open, its serous lining was gently seized and kept everted. The sponges, too, and napkins were of the finest quality. The operation, altogether, was a very tedious and trying one, requiring great patience, skill and caution in its performance; and these had their perfect work in the surgeon, who has already removed the uterus several times, partially or in its totality, by gastrotomy.

When I left Paris, the patient was reported doing well. Time is not given me to narrate what I saw, too, of Prof. Dolbeau this same day, and learned from himself the minutiæ of his new perineal dilatation as a substitute for lithotomy.

Au revoir. Your friend,

PAUL F. EVE, M. D.

---

CAIRO, EGYPT, July 30, 1873.

*Prof. E. S. Gaillard, Editor of the Richmond and Louisville Medical Journal:*

DEAR DOCTOR,—Since my arrival in Egypt, I have received letters from a large number of my medical friends in America, applying for position in the Army of the Khedive. As it is impossible to write to each one of them, I take the liberty of asking you to publish this letter, as an answer to all who have written to me on the subject.

At this time, there *are no medical vacancies in the Egyptian Army, and the Vice-Roy does not desire to engage the services of foreign surgoons.* Should there be a change in the policy of His Highness in this regard, I will announce the fact at an early day through your journal. Very truly yours,

EDWARD WARREN, M. D.,

Surgeon to the Staff of the Egyptian Army.

—*Richmond & Louisville Medical Journal.*

